# Dataset of three-dimensional traces of roads

**DOI:** 10.1038/s41597-019-0147-x

**Published:** 2019-08-05

**Authors:** Francisco Javier Ariza-López, Antonio Tomás Mozas-Calvache, Manuel Antonio Ureña-Cámara, Paula Gil de la Vega

**Affiliations:** Department of Cartographic, Geodetic Engineering and Photogrammetry, University of Jaén, Jaén, Spain

**Keywords:** Databases, Geography

## Abstract

We present a dataset consisting of three-dimensional traces, captured by Global Navigation Satellite System techniques with three-dimensional coordinates. It offers 138 traces (69 going and 69 returning), in addition to the actual mean axis of the road determined by precise surveying techniques to be used as ground truth for research activities. These data may serve as a test bed for research on data mining applications related to Global Navigation Satellite System multitraces, particularly the development and testing of algorithms intended for mining mean axis data from road multitraces. The data are suitable for the statistical analysis of both single-trace and multitrace datasets (e.g., outliers and biases).

## Abstract (summary)

**Table:** 

Design Type(s)	time series design • repeated measure design • data collection and processing objective • modeling and simulation objective
Measurement Type(s)	position
Technology Type(s)	GPS navigation system
Factor Type(s)	
Sample Characteristic(s)	Province of Jaen • road

Machine-accessible metadata file describing the reported data (ISA-Tab format)

## Background & Summary

Roads are an important component of national infrastructure and are traditionally represented in maps. Currently, road networks represent a key element of national and regional spatial databases, for example, Euroglobalmap^1^ or the transport network theme of Inspire^2^. Additionally, there are many applications for cars (e.g., TomTom™ and Here™) and for mobile devices and desktop computers (e.g., Google Maps® and OpenStreetMaps®) that offer different services (e.g., tracking, routing, and navigating) based on road networks.

The automatic generation and updating of road networks has received considerable research attention^3–8^. In this automatic environment, two processes are of great importance: the mining of all available data to derive road sections or networks^4,9–14^ and the assessment of the positional accuracy of the results using line-based methods^15–23^.

Road networks are recorded in digital spatial databases as node-edge structures, where each edge represents the axis of a road section. In the majority of cases, the edge geometry has only two-dimensional (2D) coordinates and is supported by a string of lines (polygonal), which represent the simplest geometric primitive for recording the axes of linear features. From a conventional perspective, this geometry can be generated with different technologies, for example, by a topographic or Global Navigation Satellite System (GNSS) survey or by photogrammetric or mobile mapping techniques. Data mining techniques, however, can be used at present to obtain these road axes. For example, TomTom™ and Here™ have communities of users that are allowed to upload their actual GNSS traces, and after a mining process, these traces are used to update the road database; this is known as information of communities (IC). Another possibility is the use of information known as volunteered geographical information (VGI), which is created or collected by volunteers^24^. VGI allows the generation and maintenance of bulky sets of geographic information in the form^25^ of points of interest, comments, images (photographs), navigation traces, etc. IC and VGI are similar because many contributors with a substantial number of different configurations (e.g., the branch and type of GNSS devices) are possible. Nevertheless, when IC comes from a community organized by users of a specific trademark, VGI is considered to have more variability. In the case of VGI, several GNSS trails can be downloaded from the same path on some platforms, e.g., Wikiloc (https://www.wikiloc.com/) and Wikirutas (http://www.wikirutas.es/). Nevertheless, VGI regarding paths presents several problems^26^ when trying to use it to determine path axes.

Given a dataset of multiple traces coming from IC or VGI sources, the derivation of road axes that conform to the road network structure represents a complex data mining procedure^4,7,11–13,27^. In addition, another frequent problem is the absence of ground truth with which to evaluate the geometric quality and positional accuracy of the results.

The 3D GNSS-road trace dataset that we present is the first database with multiple actual road axes captured by GNSS techniques that have three-dimensional coordinates (3D). This dataset is not VGI or IC because it has been created under a controlled design within a research project. This dataset offers 138 traces (69 going and 69 returning). The trajectories were surveyed on a set of roads that define a circular circuit with high altimetric differences, slopes and sharp curves. The actual mean axis of the road, which was determined by precise survey techniques, is supplied to be used as ground truth for research activities. Statistics about the multitraces and the axis dataset are included.

This 3D GNSS-road trace dataset could be of great help to researchers in data mining related to GNSS multitraces (e.g., algorithm development). The main application of this dataset is the development and testing of algorithms intended for mining mean axis data from road multitraces. Additionally, the dataset is suitable for the statistical analysis of single-trace and multitrace datasets, including the determination of outliers, biases and so on. The inclusion of the actual mean axis of the road determined by precise techniques allows the development of quality controls for the results. This dataset facilitates the abovementioned work because it is not necessary to invest time and money to obtain such expensive data. In addition, this dataset will facilitate comparisons with future studies and their results, which is considered extremely important for the advancement of research in this field.

## Methods

In this section, we provide a definition of the data, an explanation of the design and a description of the production methods.

### Multitraces and control axis

**Fig. 1:**
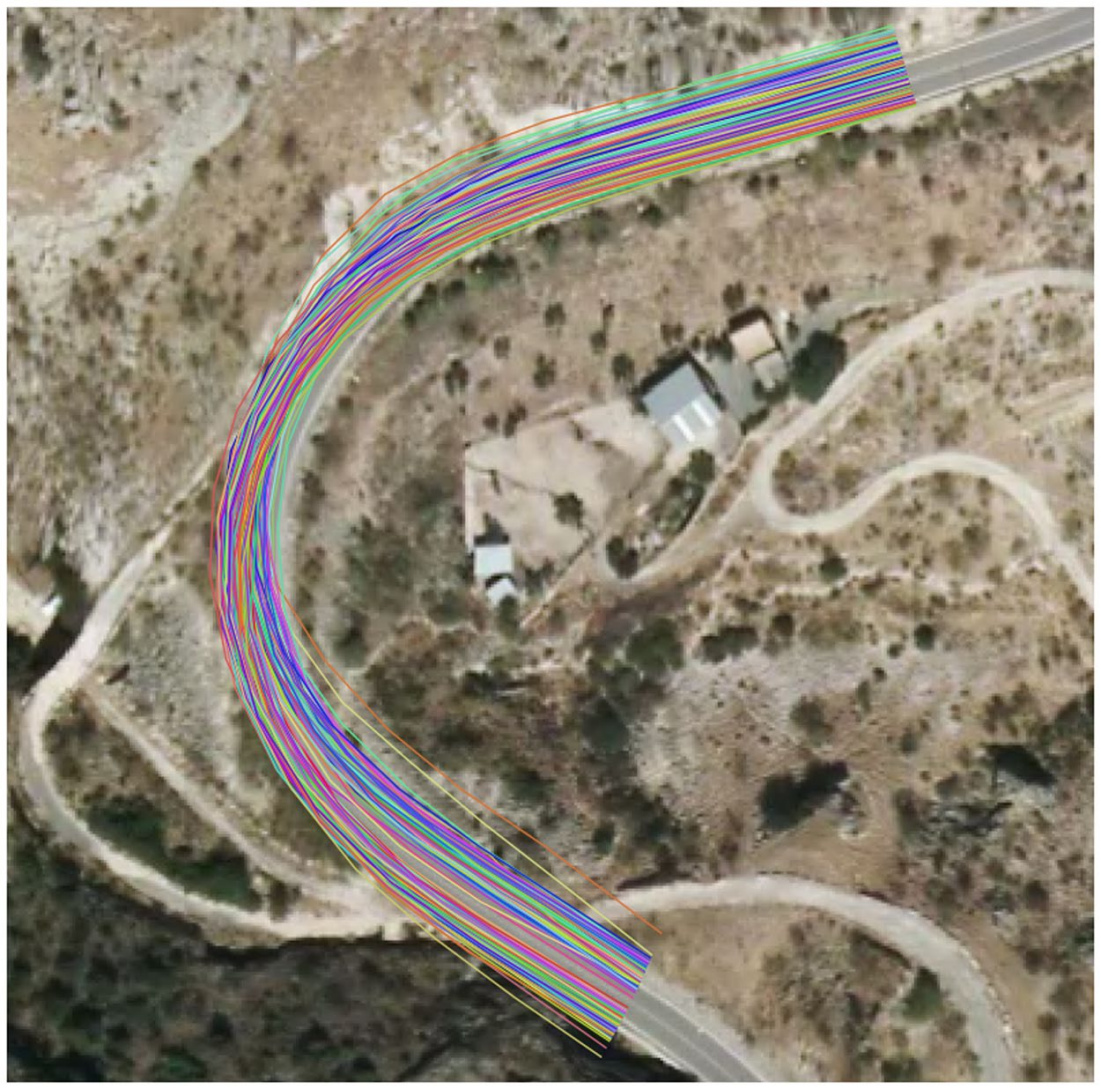
Example illustrating multitraces. Each coloured line represents a different trace. Basemap: Orthophoto from Instituto Geográfico Nacional.

A trace is a recorded GNSS path, and a multitrace set is a set of such recorded paths, as can be observed in Fig. 1.

We must define some terms to provide a better explanation of the content within the 3D GNSS-road trace dataset. The terms to be defined are GNSS trace, multitrace, axis and control axis.

**Fig. 2:**
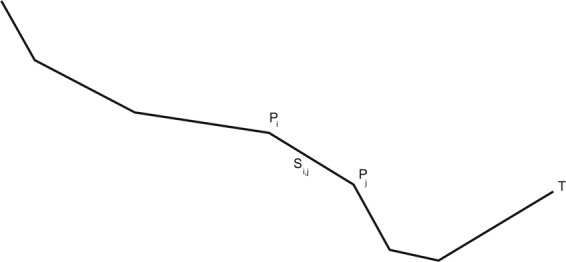
Definition of a line string *L*.

Given a geographical phenomenon such as the centreline of a road, railway, shoreline, border, river or stream, a line string *L* or polygonal representation is determined by a set {*P*_*1*_, … *P*_*n*_} of n ordered points (vertexes) that determine an ordered set *S* of n − 1 segments (S_*i*,*i*+*1*_) formed by two consecutive vertexes (*P*_*i*_, *P*_*i*+1_) (see Fig. 2). Each vertex is represented by a 3D point with coordinates {*X*, *Y*, *Z*} in a specific coordinate reference system. In an analytical way:

$$\begin{array}{c}\begin{array}{rcl}L & = & \{{S}_{0,1},{S}_{1,2},\ldots ,{S}_{n-2,n-1}\}\\  &  & {S}_{i,i+1}=\{{P}_{i},{P}_{i+1}\}\end{array}\\ {P}_{i}\left\{{X}_{i},\,{Y}_{i},\,{Z}_{i}\right\},\quad {\rm{i}}\in [0,1,\ldots ,{\rm{n}}-1]\\ {P}_{j}\left\{{X}_{j},\,{Y}_{j},\,{Z}_{j}\right\},\quad {\rm{j}}={\rm{i}}+1\end{array}$$

where:

*n*: number of vertexes.

If a line string *L* represents the path through a road section of a vehicle (e.g., a car or a motorcycle) captured by a GNSS device, *L* is called a GNSS trace (simply called a trace) and is denoted by *T*. If several traces *T* are available for the same road section, a multitrace *MT* set is established.

If a line string *L* represents the mean axis of a phenomenon (e.g., the central axis of a paved road), *L* is called the axis and is denoted by *A*. If an axis *A* is determined in such an accurate way (i.e., at least three times more accurate than an *MT* set), this axis can be used as a control axis for the *MT* set and is denoted *CA*_*MT*_.

### Design

The purpose of this dataset is two-fold; on the one hand, the dataset exists to offer a set of actual GNSS multitraces to work with and to examine all the problems corresponding to their use (omissions, outliers, bias, axis mining, etc.); on the other hand, the dataset is intended to offer an accurate mean axis for a road that can be used to control the result of mining an estimated axis from the multitrace dataset. The design covers both the area and device selection and the data processing method.

### Area selection and description

To offer an *MT* dataset characterized by sufficient complexity and variability, an appropriate study area was sought. The criteria for the selection of the study area were as follows:

- Proximity to the continuously operating GNSS reference station at the University of Jaén to obtain differential corrections for the precise GNSS survey of the accurate axis.
- Circular travel route and design to facilitate the logistics of the survey.
- Roads with little traffic to facilitate field work.
- Roads exhibiting a considerably variable slope with areas of both curved and straight segments.

**Fig. 3:**
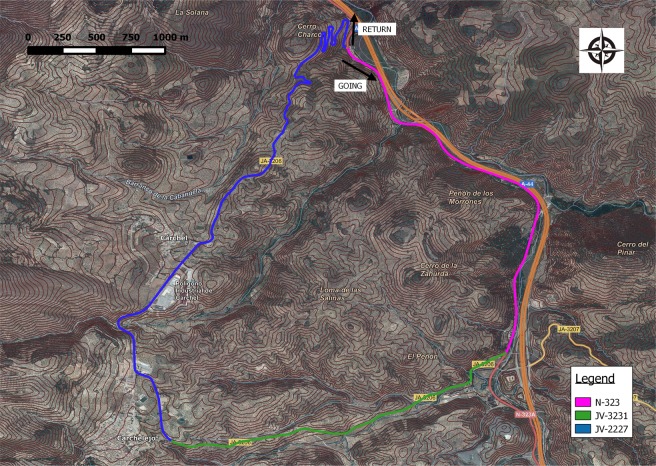
Round trip, topography and roads.

**Fig. 4:**
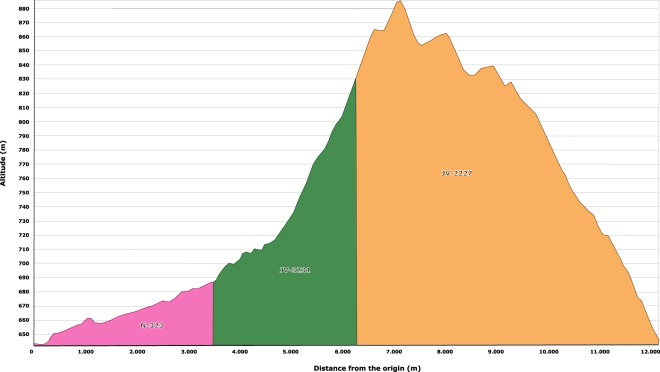
Altimetric profile.

Finally, a circular path was found on the outskirts of two small villages (Cárchel and Carchelejo) near Jaén (Spain). The total length of the path is 12.2 km, and it has a mean slope of 6%. This path is composed of three different road sections from primary and tertiary roads (Table 1). Figure 3 shows a general view of the area, and Fig. 4 presents a profile of the circular path.

**Table 1:** Definition of the linear feature.

Section	Length (km)	Mean slope
N-323	3.5	4.3%/−3.3%
JV-3231	2.8	6.4%/−4.1%
JV-2227	5.9	6.0%/−8.8%
All	12.2	6.2%/−5.7%

### Multitrace production

The *MT* dataset was captured using a Columbus V990 device (http://cbgps.com/v990/index_en.htm), which is a GNSS data logger that allows the use of a memory card to record a vast number of points and is designed for navigation and in-car applications. The accuracy specifications of the device indicate a 5.0 m circle of error probable (CEP) (95%) for non-differential GNSS applications using the Global Positioning System (GPS) and a 2.5 m CEP (95%) for differential GNSS applications using either EGNOS (European Geostationary Navigation Overlay Service) or WASS (Wide Area Augmentation System) as supplementary systems. However, the device is able to report only on the dilution of precision (DOP) of each point (no precision is offered). The device was placed on the front dashboard of a car, and the survey was performed using the non-differential configuration.

The car was driven in a normal way while taking into account all traffic signals and road conditions (e.g., slopes). A total of 69 traces were obtained in each direction (going and returning). Each trace was assigned an identifier and a label indicating whether it was a going trace or a returning trace.

### Control axis production

The centreline of a road section is the objective sought through the *MT* sets; for this goal, we need ground truth to assess the positional accuracy. This ground truth is what we call the control axis. The production of the control axis involves two main steps: first, a precise differential GNSS survey is conducted following the white roadside lines; then, the mean axis to be used as the ground truth (control axis) is calculated.

**Fig. 5:**
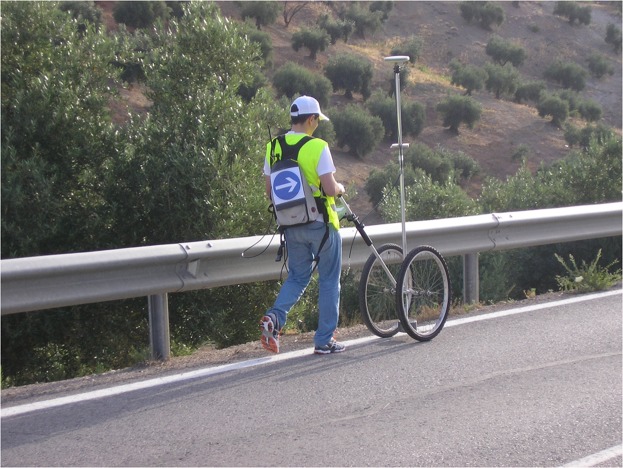
Precise GNSS survey using the Leica 1200 system.

The GNSS survey was performed using a Leica 1200, which has a horizontal accuracy of 10 mm + 1 ppm and a vertical accuracy of 20 mm + 1 ppm for kinematic surveying^28^. Post-processing was carried out using the corrections provided by the continuously operating GNSS reference station at the University of Jaén. This survey was executed on foot using the device^29^ shown in Fig. 5 that allowed the GNSS antenna to be kept vertical. The post-processed points had a planimetric precision better than 40 mm (1 sigma) and an altimetric precision better than 50 mm (1 sigma).

**Fig. 6:**
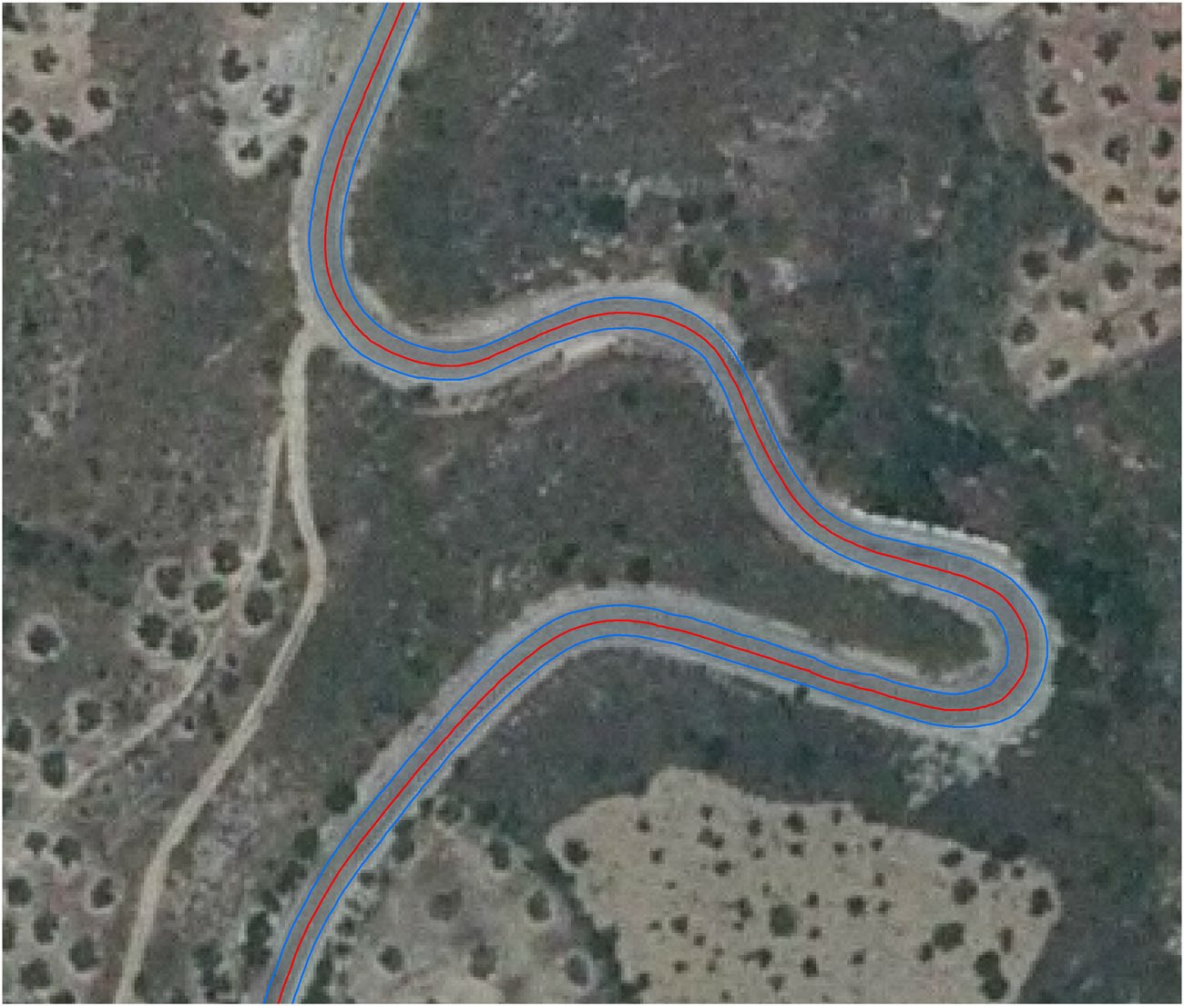
The results of the survey of the two white roadside lines (in blue) and the mean axis or control axis (in red).

Once both white roadside lines had been obtained, the mean road axis was derived using the Fréchet distance^30^ (see Gil de la Vega^31^ for more details). After this, a visual inspection of the mean axis was performed to check for the inexistence of artefacts. Figure 6 shows an example of the two white roadside lines (in blue) and the control axis (mean axis) derived (in red). A precision value was obtained for each point along the mean axis by composing the precision of the post-processed GNSS data from the survey of the two white roadside lines. The points selected for each composition were determined through an interpolation process.

## Data Records

The 3D GNSS-road trace dataset is composed of three files: two contain the spatial data, while the third file contains the metadata. The spatial data files are provided in SHP (shapefile) format^32^. The data are offered in two different coordinate reference systems (LatLon + UTM projected) to facilitate their use in research applications (the coordinate reference systems are EPSG4979 and EPSG25830, respectively). The ISO 19115-1 metadata are provided following the ISO 19115-1 standard^33^ and include the purpose, lineage and usage, in addition to many other technical characteristics. The dataset is available from Figshare^34^ as a compressed (.zip) file that contains the geospatial data in shapefile format. The fields of the SHP files and their explanations are provided in Tables 2 through 5.

**Table 2:** Attributes of traces in the multitrace dataset (EPSG: 4979).

Field	Data Type	Description
3DGRT_ID	Integer	**U**nique **i**dentifier.
3DGRT_IDT	Integer	**T**race **u**nique **i**dentifier.
3DGRT_DI	Character	**Di**rection: G = going, R = return.
3DGRT_DE	Character String	**DE**vice: Columbus_V990
3DGRT_SO	Integer	**S**equential **o**rder of point.
3DGRT_LAT	Double	**Lat**itude: decimal degrees. CRS = EPSG4979
3DGRT_LON	Double	**Lon**gitude: decimal degrees. CRS = EPSG4979
3DGRT_h	Double	Ellipsoidal **h**eight: metres. CRS = EPSG4979
3DGRT_HDOP	Double	**H**orizontal **d**ilution of **p**recision.
3DGRT_VDOP	Double	**V**ertical **d**ilution of **p**recision.

**Table 3:** Attributes of traces in the multitrace dataset (EPSG: 25830).

Field	Data Type	Description
3DGRT_ID	Integer	**U**nique **i**dentifier.
3DGRT_IDT	Integer	**T**race **u**nique **i**dentifier.
3DGRT_DI	Character	**Di**rection: G = Going, R = Return.
3DGRT_DE	Character String	**DE**vice: Columbus_V990
3DGRT_SO	Integer	**S**equential **o**rder of point.
3DGRT_X	Double	**Easting UTM**: metres. CRS = EPSG25830
3DGRT_Y	Double	**Northing UTM**: metres. CRS = EPSG25830
3DGRT_h	Double	Ellipsoidal **h**eight: metres. CRS = EPSG25830
3DGRT_HDOP	Double	**H**orizontal **d**ilution of **p**recision.
3DGRT_VDOP	Double	**V**ertical **d**ilution of **p**recision.

**Table 4:** Attributes of the control axis (EPSG: 4979).

Field	Data Type	Description
3DGRT_ID	Integer	**U**nique **i**dentifier.
3DGRT_DI	Character	**Di**rection: A = Axis
3DGRT_SO	Integer	**S**equential **o**rder of point.
3DGRT_LAT	Double	**Lat**itude: decimal degrees. CRS = EPSG4979
3DGRT_LON	Double	**Lon**gitude: decimal degrees. CRS = EPSG4979
3DGRT_h	Double	Ellipsoidal **h**eight: metres. CRS = EPSG4979
3DGRT_QP	Double	**Q**uality of **p**lanimetric position: root mean square error (RMSE) in metres
3DGRT_Qh	Double	**Q**uality of ellipsoidal **h**eight: root mean square error (RMSE) in metres
3DGRT_QPh	Double	Quality of 3D position: composition of 3DGRT_QP and 3DGRT_Qh in metres.

**Table 5:** Attributes of the control axis (EPSG: 25830).

Field	Data Type	Description
3DGRT_ID	Integer	**U**nique **i**dentifier.
3DGRT_DI	Character	**Di**rection: A = Axis
3DGRT_SO	Integer	**S**equential **o**rder of point.
3DGRT_X	Double	**Easting UTM:** metres. CRS = EPSG25830
3DGRT_Y	Double	**Northing UTM**: metres. CRS = EPSG25830
3DGRT_h	Double	Ellipsoidal **h**eight: metres. CRS = EPSG25830
3DGRT_QP	Double	**Q**uality of **p**lanimetric position: root mean square error (RMSE) in metres
3DGRT_Qh	Double	**Q**uality of ellipsoidal **h**eight: root mean square error (RMSE) in metres
3DGRT_QPh	Double	Quality of 3D position: composition of 3DGRT_QP and 3DGRT_Qh in metres.

To describe some characteristics of the dataset, some statistical information about both the *MT* set and the control axes can be seen in Table 6. Please note that the *MT* data are raw data, and for this reason, values such as the minimum distance are 0 while the slope variation rises to 1136%. These artefacts in the *MT* data have not been removed for the reason indicated in the Introduction and will be noted in the Technical Validation.

**Table 6:** Statistics of the dataset.

	*MT*	Control Axis
Number of points	111113	11304
2D distance between points [m] (min/mean/max/stdev)	0/15.05/221.32/4.63	0.01/1.07/112.14/1.64
3D distance between points [m] (min/mean/max/stdev)	0/15.07/221.32/4.62	0.01/1.08/112.14/1.64
Mean velocity [km/h]	55	—
Altimetric statistic [m]	581.02/747.62/983.02/74.51	642.95/759.95/884.90/76.35
Turning value [°]	−166.95/0.18/160.03/8.86	−59.18/−0.02/54.54/3.46
Absolute slope [%]	0.00/4.58/1136.36/6.97	0.00/4.96/40.70/3.41
HDOP (provided by GNSS) (min/mean/max/stdev)	1.1/2.1/10/0.7	—
VDOP (provided by GNSS) (min/mean/max/stdev)	0.8/1.3/10.0/0.4	—
Calculated planimetric RMSE [m] (min/mean/max/stdev)	—	0/0.02/0.73/0.05
Calculated altimetric RMSE [m] (min/mean/max/stdev)	—	0/0.02/1.16/0.10
Calculated 3D RMSE [m] (min/mean/max/stdev)	—	0/0.04/1.36/0.11

## Technical Validation

**Fig. 7:**
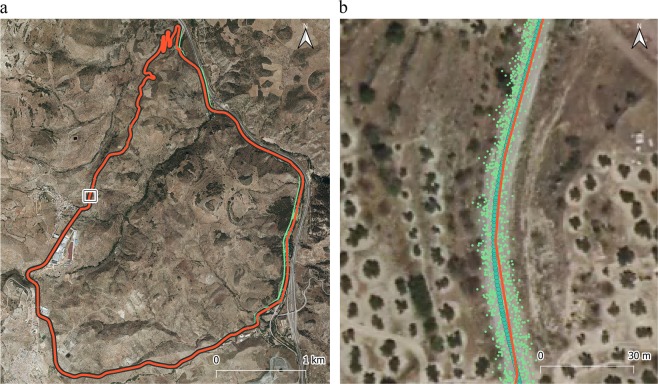
Visual test among the control axis (green dots with solid line), multitrace points (small green points) and the axis that exists on the official cartography of the IGN (red line). (**a**) General view; (**b**) detailed view. Basemap: Orthophoto from IGN (2014).

This section presents relevant information on the 3D GNSS-road trace *MT* dataset and the control axis data and processing method to assure the readability and quality of the dataset. Following the quality description framework of the ISO 19157 standard^35^, there are five categories of quality elements employed to describe the quality of geospatial data: completeness, positional accuracy, thematic accuracy, temporal quality, and logical consistency. However, neither the *MT* dataset nor the control axis data can be evaluated with respect to temporal quality because the complete dataset was surveyed over a very short period of time, and thus, no temporal quality aspect is involved or relevant to the whole dataset. However, we can provide a discussion about the other four quality elements for the *MT* dataset and for the control axis:

1. Completeness. Raw GNSS data points from the data logger have been provided. These data are offered without processing to ensure the presence of all possible artefacts that may occur in any survey. This is of great interest for future research (e.g., the treatment of outliers and errors). However, the *MT* points were inspected visually, as can be seen from the general view in Fig. 7a and the detailed view in Fig. 7b. In addition, the control axis has been visually inspected by three operators and is complete (Fig. 8).

**Fig. 8:**
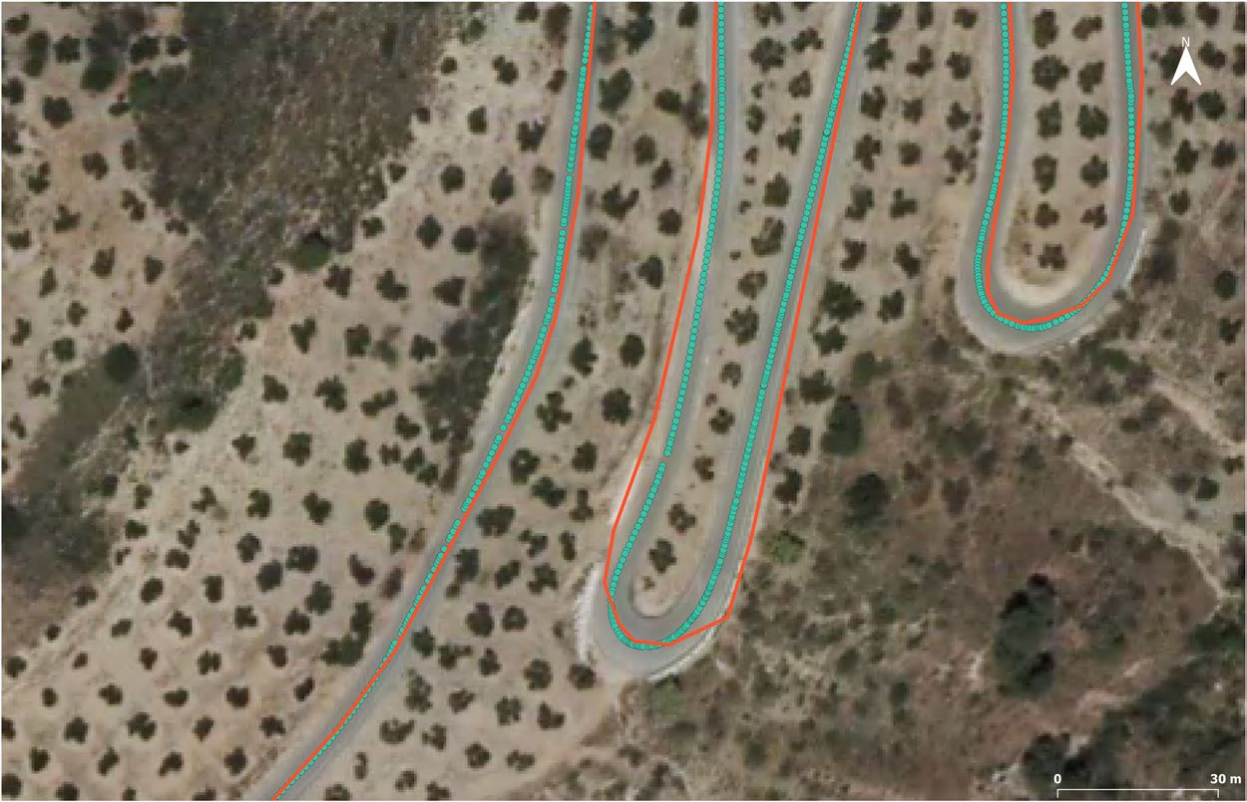
Details of the differences between the control axis (green dots with solid line) and the axis that exists on the official cartography of the IGN (red line) in a sinuous section. Basemap: Orthophoto from IGN (2014).
2. Positional accuracy. The accuracy of the raw GNSS data points has been provided as registered by the data logger. With respect to the control axis, the surveying device and processing method guarantee a positional accuracy that is at least three times greater than the positional accuracy of raw GNSS data; hence, the raw data can be used as a control axis. Moreover, the per point accuracy has been provided as the RMSE compositions of the points used to define each vertex of the mean axis. In addition, a visual control has been executed, and a complete comparison of the mean axis with an independent official source (at a 1:25000 scale) (Fig. 8) clearly shows that the provided control axis boasts a better performance.
3. Thematic accuracy. The only thematic attribute that appears in this dataset is the 3DGRT_DI field. The assignment of all the values has been reviewed and found to be correct in 100% of the cases.
4. Logical consistency. Among all the logical consistency aspects of geospatial data, only the format consistency can be checked in this dataset. To test the Shapefile format, all files were loaded using ArcMap from ESRI™ and GDAL (http://gdal.org). Both pieces of software loaded the Shapefiles, including the height values from the geometry, thereby validating the file structure and compatibility. However, we have included the geometry tuple in the attributes of each feature to ensure the ability to analyse this third coordinate.

## Usage Notes

The dataset is distributed as Shapefiles that contain the data organized as described in Tables 2 through 5. Shapefiles are the standard for exchanging and storing spatial data. Many Geographic Information System tools (e.g., ArcGIS®, QGIS®, and GRASS®) are able to load such files. The use of scripting languages (e.g., Python) within these software tools can be of great help for the processing of multitrace datasets because there are no standard capabilities for such types of data. The GDAL library for R (https://www.R-project.org/) allows access to the data and the use of many other R packages with powerful capabilities for dealing with trace and multitrace datasets.

The 3D GNSS-road trace dataset is available for free use/reuse. There are no restrictions to support the widest possible use. The main application of this dataset is the development and testing of algorithms intended for mining mean axis data from road multitraces^4,7,11–13,27,36^. Multitraces are provided as raw data, and thus, this dataset is suitable for the statistical analysis of both single-trace and multitrace datasets, including the determination of outliers and biases and the development and testing of filtering algorithms focused on problems pertaining to traces^31,37^. Finally, the number of traces allows the use of simulation procedures (Monte Carlo, Bootstrap, etc.) to derive and estimate the distributions of some characteristics; for instance, these simulation techniques can be applied to determine the adequate sample size.

## ISA-Tab metadata file

Download metadata file
